# Image2Flow: A proof-of-concept hybrid image and graph convolutional neural network for rapid patient-specific pulmonary artery segmentation and CFD flow field calculation from 3D cardiac MRI data

**DOI:** 10.1371/journal.pcbi.1012231

**Published:** 2024-06-20

**Authors:** Tina Yao, Endrit Pajaziti, Michael Quail, Silvia Schievano, Jennifer Steeden, Vivek Muthurangu

**Affiliations:** Institute of Cardiovascular Science, University College London, London, United Kingdom; Stanford University, UNITED STATES

## Abstract

Computational fluid dynamics (CFD) can be used for non-invasive evaluation of hemodynamics. However, its routine use is limited by labor-intensive manual segmentation, CFD mesh creation, and time-consuming simulation. This study aims to train a deep learning model to both generate patient-specific volume-meshes of the pulmonary artery from 3D cardiac MRI data and directly estimate CFD flow fields. This proof-of-concept study used 135 3D cardiac MRIs from both a public and private dataset. The pulmonary arteries in the MRIs were manually segmented and converted into volume-meshes. CFD simulations were performed on ground truth meshes and interpolated onto point-point correspondent meshes to create the ground truth dataset. The dataset was split 110/10/15 for training, validation, and testing. Image2Flow, a hybrid image and graph convolutional neural network, was trained to transform a pulmonary artery template to patient-specific anatomy and CFD values, taking a specific inlet velocity as an additional input. Image2Flow was evaluated in terms of segmentation, and the accuracy of predicted CFD was assessed using node-wise comparisons. In addition, the ability of Image2Flow to respond to increasing inlet velocities was also evaluated. Image2Flow achieved excellent segmentation accuracy with a median Dice score of 0.91 (IQR: 0.86–0.92). The median node-wise normalized absolute error for pressure and velocity magnitude was 11.75% (IQR: 9.60–15.30%) and 9.90% (IQR: 8.47–11.90), respectively. Image2Flow also showed an expected response to increased inlet velocities with increasing pressure and velocity values. This proof-of-concept study has shown that it is possible to simultaneously perform patient-specific volume-mesh based segmentation and pressure and flow field estimation using Image2Flow. Image2Flow completes segmentation and CFD in ~330ms, which is ~5000 times faster than manual methods, making it more feasible in a clinical environment.

## Author summary

Computational fluid dynamics is an engineering tool that can be used in a clinical setting to non-invasively model blood flow through blood vessels, such as the pulmonary artery. This information can be used to inform treatment planning for patients, especially those with cardiovascular conditions. However, its routine use is limited by a labor-intensive process, requiring substantial expertise and computational resources. Recently, machine learning has offered solutions to automate parts of this process, but no single model has addressed the entire workflow.

Therefore, we created Image2Flow, a machine learning model capable of generating a patient-specific representation of a pulmonary artery while predicting blood flow through the vessel. Image2Flow can generate highly accurate pulmonary artery representations and reasonably accurate blood flow predictions. Notably, it performs this whole process in 330ms, which is ~5000x faster than the manual methods. With its speed and accuracy, Image2Flow holds substantial promise for facilitating computational fluid dynamics in clinical settings.

## Introduction

Computational fluid dynamics (CFD) can aid the management of congenital heart disease (CHD) [1–3] through non-invasive estimation of hemodynamics (e.g. pressure gradients) and prediction of hemodynamic response to therapy. Several cardiovascular conditions have already been investigated using CFD, including coronary artery anomalies [4], aortic coarctation [5], tetralogy of Fallot [6], and univentricular hearts [7,8]. However, translation of CFD into the clinical environment is currently limited by: i) Time-consuming manual image segmentation [9], ii) CFD mesh generation, which is complex and often requires engineering expertise [10], and iii) The computationally intensive nature of CFD simulations, which results in long computation times [11,12].

Recently, deep learning (DL) approaches have been shown to provide accurate voxel-wise segmentation of computed tomography and magnetic resonance imaging (MRI) data [13]. Importantly, meshes derived from these DL segmentations provide comparable CFD results to human segmentations [14]. Nonetheless, voxel-wise segmentation can result in misclassified regions and other anatomical inconsistencies. Furthermore, mesh generation is still required, which requires some expertise and can be time-consuming. Thus, graph-based neural networks that directly generate surface-meshes from 3D image data have also been investigated [15,16]. Notably, these mesh-based models outperform voxel-based methods in terms of both accuracy and the direct generation of smooth, CFD "simulation-suitable" meshes [17,18].

Although speeding up segmentation/mesh generation removes one of the barriers to clinical translation, CFD is still limited by long computation times. There have been studies that have used DL to speed up CFD. These include using anatomical shape descriptors as machine learning model inputs [19–22], as well as point-cloud [23,24] and graph-based [25] methods that act directly on meshes. However, there has been limited research into using DL to automate both the segmentation and CFD simulation process in a single model.

In this proof-of-concept study, we propose to build on previous graph-based methods to simultaneously generate volume-meshes from 3D MRI and directly estimate pressure and velocity at each vertex. The aims of this study were to: i) Develop a DL model capable of taking a 3D cardiac MRI, creating a volume mesh reconstruction of the pulmonary artery, and predicting pointwise CFD pressure and velocity, ii) Evaluate segmentation accuracy by comparison with previous DL methods and, iii) Evaluate the proposed DL model by comparing point-wise DL-predicted CFD pressure and flow with results obtained from a conventional CFD solver.

## Materials and methods

### Ethics statement

The collection of the private dataset used in this study conformed to the principles of the Declaration of Helsinki and was approved by the UK National Health Service, Health Research Authority, Research Ethics Committee and written informed consent was obtained for all subjects (Code: 06/Q0508/124).

### Image2Flow model architecture

**Fig 1:**
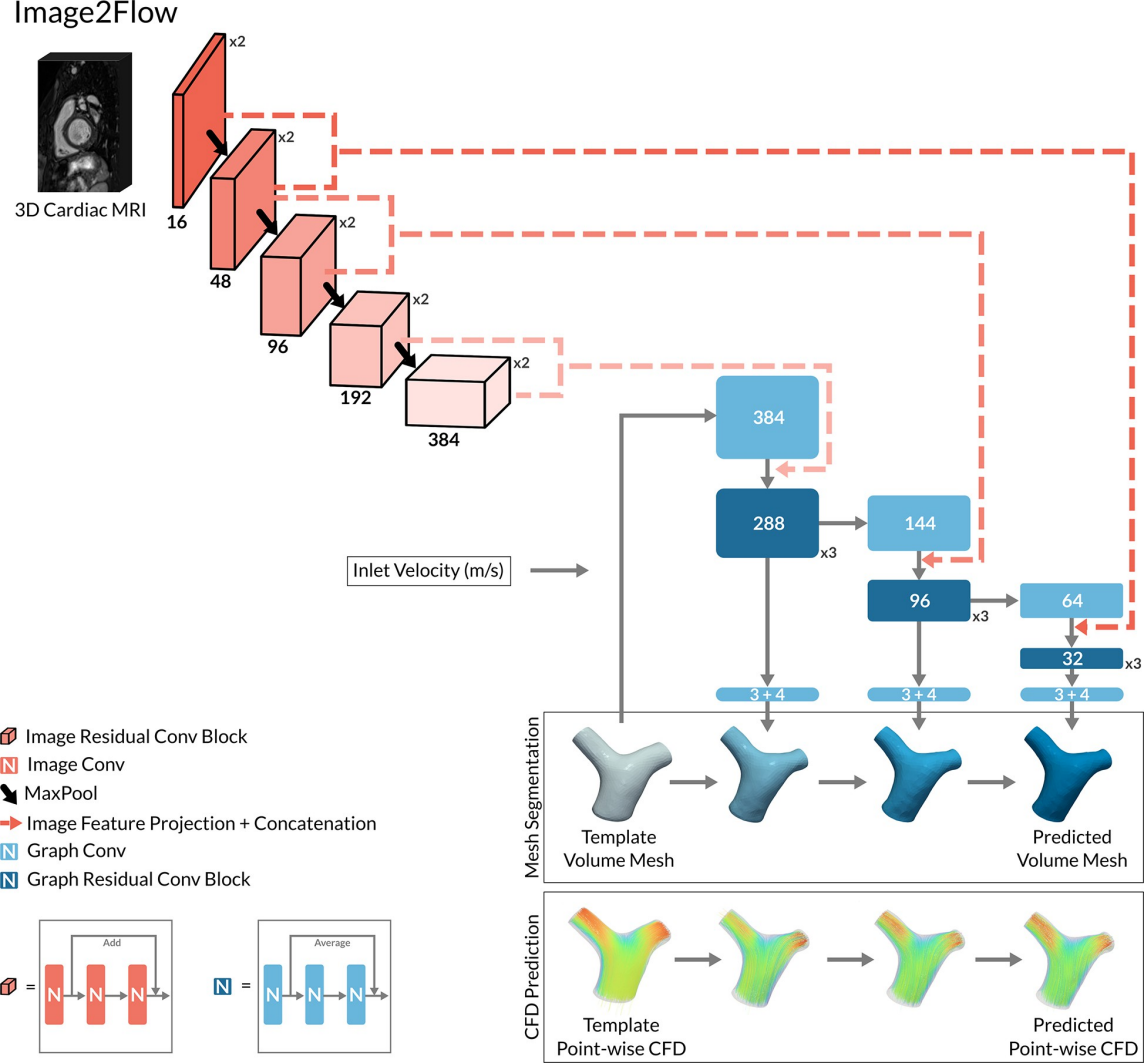
The model architecture of Image2Flow. The hybrid image and graph convolutional neural network architecture of Image2Flow. It takes as input a 3D cardiac MRI and a template volume mesh of a pulmonary artery. It outputs the patient-specific pulmonary artery mesh with associated pressure and flow at each node.

Our DL model—Image2Flow (Fig 1)—builds on the previously described MeshDeformNet, which combines an image convolutional encoding arm with a graph convolutional template transformation arm [16] to perform surface-based segmentation. Image2Flow improves on this by simultaneously performing volume-mesh segmentation and predicting pressure and flow at each vertex, leveraging an inputted patient-specific inlet flow rate.

#### Image encoding arm

Image2Flow starts with an image encoding arm that takes a 3D MRI whole heart dataset as an input. The image encoding arm consists of five convolutional levels, each containing two convolutional residual blocks followed by a maxpool layer (Fig 1). Each convolutional residual block includes three sets of 3x3x3 convolution filter layers (followed by instance normalization and LeakyReLU activation), as well as a spatial dropout layer and a residual connection. The number of convolutional filters increases at each level (16, 48, 96, 192, and 384).

#### Graph transformation arm

The patient-specific output of Image2Flow was generated using the graph transformation arm, which utilizes features from the image encoding arm to transform a template mesh. The template volume-mesh was represented by a graph containing 10,998 nodes each with seven features (x-y-z coordinates, pressure, and flow), (Fig 1). The graph transformation arm comprised of three sequential transformation branches, each performing graph convolutions that successively convert the template into a patient-specific volume-mesh with estimated pressures and velocities at each vertex. We employed first-order Chebyshev convolutional layers from the Spektral library [26] for all graph convolutions.

The model’s initial graph convolution operated on the x-y-z coordinates of the template mesh as well as the input inlet velocity, which was incorporated by concatenating the velocity value to each node in the template mesh. Each subsequent graph transformation branch included the following steps: i) a graph convolution layer that increased/decreased the number of graph features to better match the number of image features in the corresponding levels of the image encoding arm, ii) projection of image features from two levels of the image encoding arm onto mesh nodes via trilinear interpolation, iii) concatenation of the extracted image features with graph features, iv) processing of concatenated features through a series of three graph residual convolutional blocks, and v) a bottleneck graph convolutional layer producing the transformation from the template mesh in terms of nodal x-y-z coordinates, pressure, and velocities (in x-y-z directions). Each graph convolution residual block consists of three graph convolutional layers (followed by instance normalization and LeakyReLU activation) and residual connection (Fig 1).

### Image data and processing

The training/validation/testing image dataset consisted of 135 cardiac triggered, respiratory navigated, non-contrast 3D whole-heart, balanced steady-state free precession (WH-bSSFP) acquisitions. Of the 135 datasets, 84 datasets were collected from previously scanned children and adults with pediatric heart disease or biventricular congenital heart disease (excluding patients with pulmonary artery stents, or discontinuous/unifocalized pulmonary arteries) as previously described [14]. This included 37 patients (44%) with lesions affecting the pulmonary arteries (e.g. tetralogy of Fallot, shunts, pulmonary stenosis, and repaired transposition of the great arteries). The remaining 51 datasets were derived from the public multi-modality whole heart segmentation (MMWHS) challenge data [27]. The original MMWHS data had 60 cases, but 9 datasets were excluded due to poor pulmonary artery definition.

All data were acquired with a 1.5T field strength. The private dataset exclusively comprised scans from Siemens scanners, while the public dataset included a combination of Siemens and Phillips scanned data. In the private dataset, scans were acquired isotropically with a voxel size of 1.6 mm. However, in the public dataset, voxel sizes were ~0.8–1.0 mm in-plane and ~1.0–1.6 mm through-plane. The total dataset was divided into 110/10/15 for training/validation/testing. The training set comprised a mix of private and public data, whereas the validation and test set exclusively used images randomly selected from the public dataset to better enable public evaluation. This was a different split used in the original MMWHS challenge (where 40 cases were used for testing), as more of the MMWHS data was needed for training due to the complexity of the Image2Flow problem. This was possible because we performed our own segmentations on both the private and MMWHS datasets and were therefore not limited to only using MMWHS data with segmentations (n = 20) for training.

Reference standard conventional segmentation of the pulmonary arteries was performed by a single observer (20 years’ experience in cardiac MRI post-processing) using a semi-automatic technique with manual correction (Plug-ins created in Horos v4.0, Horosproject.org, Maryland, USA). Initial segmentation was done using the fast level-set method [25]. This required the user to: i) set a threshold, ii) place seeds in the vessel of interest and iii) add blocking regions to prevent segmentation of unwanted structures. Manual correction of this initial segmentation was always required to remove unwanted structures, and this was performed using manual volume subtraction method in Horos. The WH-bSSFP data and their corresponding pulmonary artery masks were spline interpolated to create isotropic volumes with a voxel size of 1.0 mm. Both the interpolated image and mask data were centered around the position of the pulmonary artery and cropped to a 128x128x128 matrix.

#### Point-point volume mesh generation

**Fig 2:**
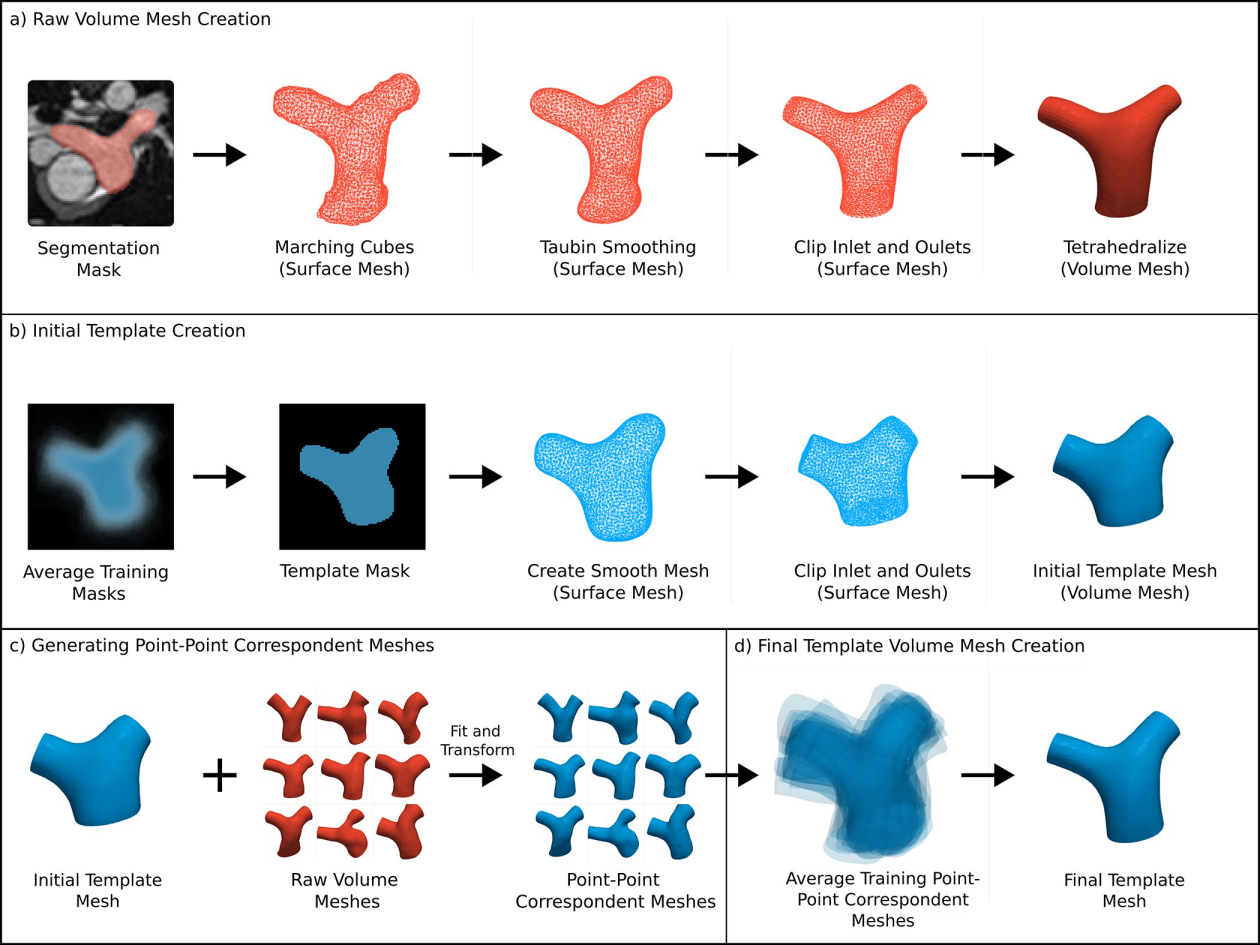
A schematic of the steps involved in point-point correspondent volume mesh generation. (A) raw patient-specific volume mesh created from manual segmentation, (B) initial template volume mesh creation, (C) point-point correspondent volume mesh generation by transforming the initial template, (D) final template volume mesh creation by averaging the point-point correspondent meshes of the training data. Red indicates non-corresponding meshes and blue represents corresponding meshes. The wireframe rendering denotes surface meshes, while the solid rendering denotes volume meshes.

A prerequisite of Image2Flow was that all the patient-specific volume-meshes (and the template mesh) contained the same number of nodes with point-point correspondence. This enabled point-wise calculation of CFD losses during training, which was necessary to ensure accurate pressure and velocity estimation. This was achieved in a multi-step process shown in Fig 2 and described below.

In the first step (Fig 2A), raw patient-specific surface-meshes were generated from the segmentation masks using the marching cubes algorithm with subsequent smoothing. The raw surfaces were then manually clipped at the sino-tubular junction (inlet) and at the hilar branches of the pulmonary arteries (outlets). The clipped patient-specific surface-meshes were defined as the ground truth (GT) surfaces. They were then converted into raw tetrahedral volume meshes using the TetGen in Vascular Modeling Toolkit (VMTK) [28]. To achieve approximate uniformity across meshes, the target edge length parameter for converting the surfaces into tetrahedral volume-meshes was adjusted iteratively until each mesh consisted of approximately ~11,000 (range: 10,890–11,922) nodes.

In the second step (Fig 2B), an initial pulmonary artery template was generated by averaging the segmentation masks from the training data and applying morphological image operations to generate a more consistent average template mask. The template mask was converted into a surface and clipped at inlet and outlets, from which a volume mesh was generated with 10,998 nodes using TetGen.

In the third step (Fig 2C), the initial template volume-mesh (generated in step 2) was deformed to match the patient-specific raw volume meshes. This resulted in patient-specific volume-meshes that had the same number of nodes, all in point-point correspondence. The deformation process consisted of training and fitting a simplified version of our Image2Flow model (excluding blood pressure and flow features and CFD losses) to all datasets. The average symmetric surface distance (ASSD) and Hausdorff distance (HD) between the ground truth surfaces and the surfaces extracted from the point-point correspondent meshes were 0.173 (0.150–0.200) and 1.718 (1.500–1.940), respectively, The Dice score between the original masks and masks derived the point-point correspondent meshes was 0.985 (0.983–0.986). These results demonstrated the robustness of the deformation process.

In the last step (Fig 2D), a final template mesh was created by averaging the node positions of the patient-specific point correspondent training meshes (generated in step 3). This final template mesh more accurately represented the mean shape of the pulmonary artery than the initial template mesh (from step 1). Centering of both templates within the distribution of pulmonary artery anatomies was done to minimize the maximum spatial deformation required to align the template with anatomies at the edge of distribution.

### Computational fluid dynamics (CFD) simulations

Neither the raw nor point-point correspondent volume-meshes were suitable for CFD computation as finer meshes and flow extensions are required. Therefore, the GT surfaces were also used to create CFD-suitable volume meshes. Firstly, using VMTK, 40mm flow extensions were added to the clipped inlet and outlets of the surface, to create flat and circular cross-sections to achieve a uniform velocity profile. Subsequently, the extended surfaces were transformed into high-resolution volume meshes using TetGen, each containing an average of approximately 500,000 tetrahedral elements. The number of tetrahedral elements was chosen after sensitivity analysis (see S1 Fig).

CFD simulations were conducted across the refined meshes for all datasets (including the template) using Fluent (Ansys, Pennsylvania, USA). All simulations used a steady inlet velocity, with the gauge pressures at the outlets set to 0Pa. For the training data, four separate CFD simulations were performed on each mesh dataset using random inlet velocities as a form of data augmentation. The velocities were chosen from a normal distribution with a mean of 0.2 m/s (average velocity through the PA over the cardiac cycle based on the higher range of normal [29]) and a standard deviation of 0.05m/s. For the test data, two sets of simulations were performed related to the different evaluations described below. In the first set, a single random inlet velocity was chosen from the same distribution as the training (0.2±0.05m/s) for testing the accuracy of Image2Flow. In the second set, three further CFD simulations were performed on each test mesh with inlet velocities of 0.1, 0.2, and 0.3 m/s to test the ability of Image2Flow to respond to increasing flow rates. A uniform set of blood properties was applied, assuming blood to be a Newtonian fluid with a density of 1060 kg/m^3^ and a viscosity of 0.04 Pa·s. Additionally, laminar, steady-state flow conditions were employed, and standard non-slip conditions were maintained at the wall boundaries.

CFD simulations were performed on the meshes lacking point-point correspondence and with inconsistent numbers of points. Therefore, the CFD results required interpolation onto the point-correspondent meshes (including the template) for pressure/flow data alignment across cases, using the method described by Pajaziti et al. [19]. The accuracy of the pressure and flow values interpolation was 0.02% (-0.3–0.2) and -0.02% (-0.2–0.2) for pressure and velocity.

### Model training

Image2Flow was trained with a NVIDIA GeForce RTX 3090 GPU card (24 GB RAM). Due to the highly skewed pressure data within and between patients, we applied a cube root transformation to normalize the distribution. Pressure (after cube rooting) and flow were then scaled by subtracting the mean and dividing by the standard deviation. After inference, the predicted pressure was cubed again to preserve the original data scale. Our model was trained with several losses to optimize segmentation accuracy, volume-mesh quality and CFD pressure/flow prediction, as described below. At each epoch, one of the four CFD simulations previously created with different inlet velocities was randomly chosen to be used for training. On the other hand, all four CFD simulations were used for validation during training. Varying the inlet velocity while keeping the geometry the same is effectively augmentation, which allows the model to learn how to generalize to different inlet velocities.

#### Point loss

A Chamfer loss was used to minimize the point-wise distance between the template PA mesh and the ground truth. We applied two Chamfer losses: one for all points (ℒ_Point_) within the mesh and another specifically for surface points (ℒ_Point,S_):

$$L_{Point}(P,G)=\sum_{p\in P}\underset{g\in G}{min}{\left\|p-q\right\|}_{2}^{2}+\sum_{g\in G}\underset{p\in P}{min}{\left\|p-q\right\|}_{2}^{2}$$

Where ***p*** and ***g*** are vertices in the point clouds (or surface point clouds) of the predicted and ground truth volume meshes ***P*** and ***G***.

#### Edge length deviation loss

We define the edge length deviation as the mean edge length in the mesh relative to their standard deviation. This is a measure of the consistency of the edge lengths throughout the mesh. This loss ensures that all the edges are approximately uniform in length and therefore the points in the template mesh translate uniformly. We applied two edge length deviation losses: one for all edges within the mesh (ℒ_Edge_) and another specifically for edges on the surface (ℒ_Edge,S_), which ensures a smooth mesh surface:

$$L_{Edge}(P)=\frac{\sigma_{Edge}}{\mu_{Edge}}$$

Where *μ*_*Edge*_ and *σ*_*Edge*_ represents the mean and standard deviation of the edge lengths (or surface edge lengths) in predicted volume mesh ***P***.

#### Aspect ratio loss

Aspect ratio is a key measure of volume mesh quality, representing the ratio of the longest to the shortest edge in each tetrahedral element. Incorporating the aspect ratio loss (ℒ_Aspect_) guarantees low-skew, consistent tetrahedral elements in predicted volume meshes and prevents self-intersecting faces:

$$L_{Aspect}(P)=\frac{1}{\left|C\right|}\sum_{C_{j}\in C}\frac{\underset{\forall v_{j}\neq v_{k}\epsilon c_{j}}{max}{\left\|v_{j}-v_{k}\right\|}_{2}}{\underset{\forall v_{j}\neq v_{k}\epsilon c_{j}}{min}{\left\|v_{j}-v_{k}\right\|}_{2}}$$

Where *C*_*j*_ represents the *j*th tetrahedral cell element in the set of tetrahedral elements, C, within the predicted volume mesh ***P***. The set of vertices in *C*_*j*_ is denoted ***c***_*j*_, and **v**_j_, **v**_k_ represent vertices in ***c***_*j*_.

#### Cap coplanar loss

Kong et al. outlined the “cap coplanar loss”, which ensures flat surfaces at the inlets and outlets of a mesh, which is desirable for CFD simulation. [17] This loss (ℒ_Cap_) minimized the normals to the faces on the cap, aligning the surface faces in the same direction.


$$L_{Cap}(P)=\sum_{j=1}^{3}\sum_{{k=F}_{j}}{\left\|n_{k}-\frac{1}{\left|F_{j}\right|}\sum_{k=F_{j}}n_{k}\right\|}_{2}^{2}$$

Where *F*_*j*_ is the mesh faces of the *j*th cap in the predicted volume mesh ***P***. There are three caps, representing the inlet and the two outlets. ***n***_*k*_ is the normal vector of the *j*th face of F_*j*._

#### CFD loss

By registering the ground truth meshes with the template meshes, we established point correspondence across all meshes. Consequently, when we interpolated CFD results onto these meshes, the blood pressures and flows also aligned. This alignment enabled the calculation of the mean absolute error loss, between ground truth and predicted pressure and velocity values (ℒ_CFD_). Standardization of pressure and flow values ensured they followed a similar scale, enabling their combination into a single CFD loss through summation.


$$L_{CFD}(P,G)=\frac{1}{N}\sum_{j=1}^{N}\left|p_{j}-g_{j}\right|$$

Where ***p***_*j*_ and ***g***_*j*_ represent the pressure and flow value at the vertex *j* of the volume meshes ***P*** and ***G***.

#### Total loss

At each of the three transformation branches, the model generated a mesh with associated pointwise CFD pressure and flow. This allowed calculation of mesh loss (ℒ_mesh_) for each transformation branch by combination of the losses:

$$L_{mesh}=\lambda_{1}(L_{point}+L_{point,S})+\lambda_{2}(L_{Edge}+L_{Edge,S})+\lambda_{3}L_{Aspect}+\lambda_{4}L_{Cap}+\lambda_{5}L_{CFD}$$

The individual losses were weighted empirically (λ_1_ = 1, λ_2_ = 0.1, λ_3_ = 0.5, λ_4_ = 0.05 and λ_5_ = 30) to create a high mesh quality, smooth, and accurate segmentation with flat inlet and outlets while also achieving the highest pressure/flow accuracy. The total loss is the sum of losses of the three transformation branches:

$$L_{Total}=\sum_{i=1}^{3}L_{Mesh}(P^{i},G)$$

Where ***P*** *and* ***G*** are the predicted and ground truth meshes, respectively.

#### Inference

At inference, 3D WH-bSSFP images from 15 unseen cases were inputted into the Image2Flow. The outputs were volume-meshes with each vertex associated with pressure and velocity values. In addition, to better understand the origin of any errors, the output volume mesh segmentations from Image2Flow (without pressure and velocity values) were also used to perform conventional CFD as previously described (CFD_DL-seg_). Inference time was measured as the time for prediction on a NVIDIA GeForce RTX 3090 GPU card (24GB RAM).

### Evaluation

#### Segmentation accuracy

Segmentation accuracy was assessed using Dice score, ASSD, and Hausdorff distance (HD). For benchmarking, we compared Image2Flow with our implementation of the MeshDeformNet [16], and a 3D UNet model as described by Montalt-Tordera et al. [14]. Our ‘MeshDeformNet’ is essentially the surface-mesh version of Image2Flow, employing the same losses except for the aspect ratio loss, which is specific to volume-meshes. The surface-meshes that were created had the same number of nodes as the volume-meshes (10,998).

As Dice score compares segmentation masks, we first converted the meshes predicted by Image2Flow and ‘MeshDeformNet’ into binary masks (the 3D UNet outputs were already binary mask). The Dice score was then calculated by comparison with the ground truth manual segmentation masks. As ASSD and HD are both computed on surfaces, the segmentation masks from the 3D UNet were first converted into surfaces and the surfaces were extracted from the Image2Flow volume-meshes (the ‘MeshDeformNet’ outputs were already surfaces). The ASSD and HD were then calculated by comparison with the ground truth surfaces.

#### CFD accuracy

We evaluated the predicted CFD results derived using Image2Flow (CFD_I2F_) by comparing the data with the ground truth CFD results interpolated onto the point-point correspondent meshes (CFD_GT_) in the following ways:

*Pressure and velocity error*: At each node, we calculated the normalized absolute error (NAE) for pressure, x-y-z velocity components and velocity magnitude as previously described [19]. The subject level mean normalized absolute error (MNAE_s_) was calculated as the average NAE value across all nodes in the mesh for each test subject. We also calculated a mean nodal error (MNAE_n_) by averaging the NAE value for each node across all test subjects. This was used to evaluate the prediction error with respect to the relative position of the node in the mesh.

*Effect of varying inlet velocity*: To evaluate Image2Flow’s ability to respond to varying flow rates, we compared the model performance for inlet velocities of 0.1, 0.2 and 0.3 m/s for each subject in the test data.

*Comparison with CFD simulations performed on DL segmentation*: The Image2Flow CFD predictions were compared to conventional CFD simulations performed using the anatomical meshes produced by Image2Flow (CFD_DL-seg_). This was done to evaluate the separate contributions of segmentation and CFD prediction errors by calculating the subject-level difference in MNAE_s_ between CFD_I2F_ and CFD_DL-seg_.

#### Statistics

Continuous variables are presented as median (interquartile range). Bland-Altman analysis evaluated bias and limits of agreement for pressure and flow predictions. The Wilcoxon signed-rank test compared segmentation performance between Image2Flow, ’MeshDeformNet’ and the 3D UNet. Conover’s test was used as a post-hoc comparisons test for the MNAE_s_ error for the three varying input inlet velocities for the test set geometries, after a Friedman test showed a significant difference between the results. The one-sample Wilcoxon signed rank test was used to compare the difference in MNAE_s_ values between CFD_I2F_ and CFD_DL-seg_ to ascertain if the median difference was significantly different to 0. The Wilcoxon signed-rank tests were used as the differences between the measurements were not normally distributed (evaluated using the Shapiro-Wilk test). P-values less than 0.05 were considered statistically significant.

## Results

### Feasibility

Image2Flow was able to successfully generate patient-specific volume-meshes with pressure and flow estimations for all subjects. The inference time was approximately 328ms per dataset. This represents a speed-up of ~5000x, with conventional segmentation, processing and simulation taking ~25 minutes in total (segmentation: 15min, mesh generation: 5min, CFD simulation: 5min).

### Segmentation accuracy

**Fig 3:**
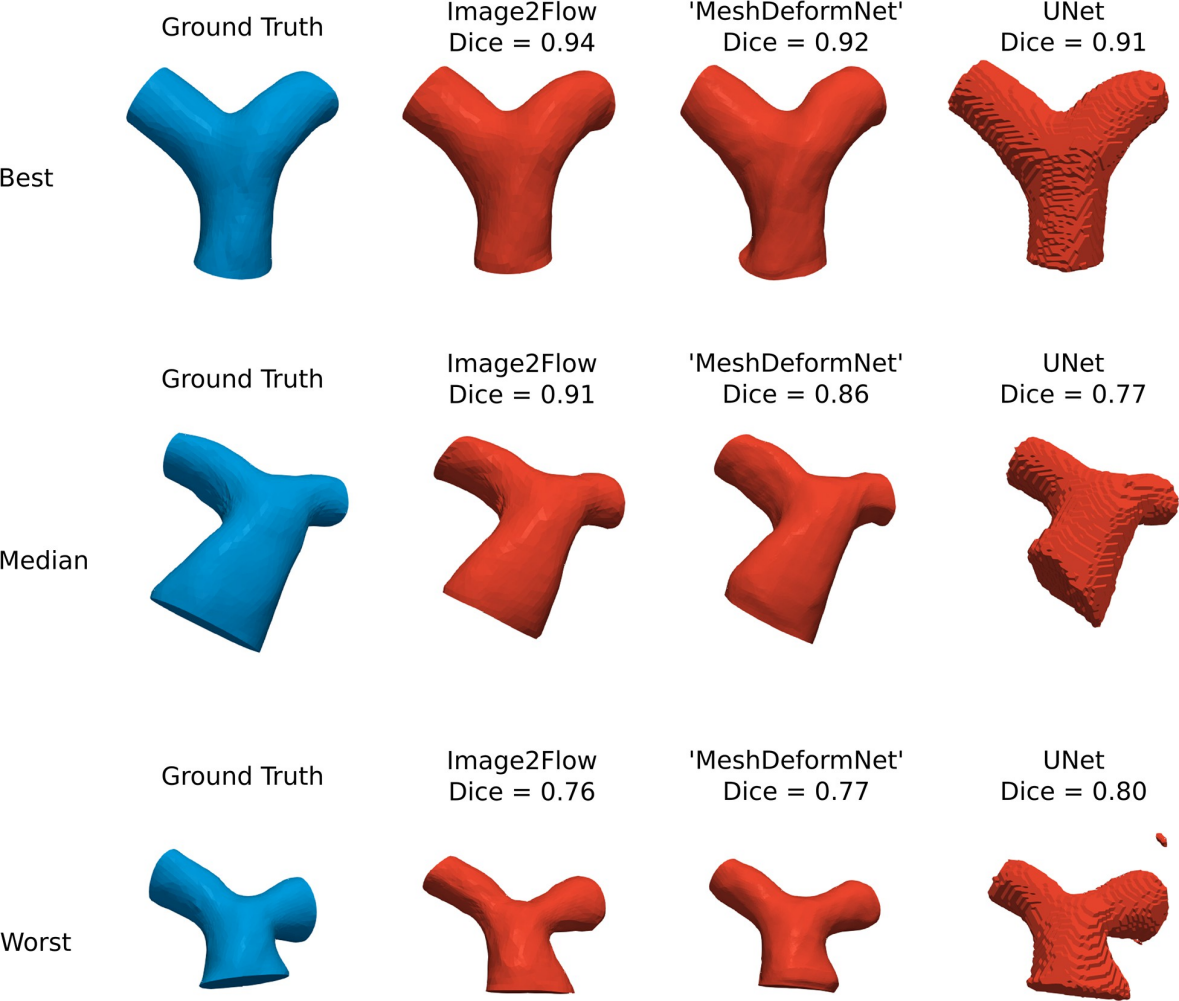
Segmentation accuracy. The best, median and worst Image2Flow segmentations compared to ‘MeshDeformNet’ and a 3D UNet.

Fig 3 shows the best, median and worst Image2Flow segmentations (in terms of Dice score) and compares with the ’MeshDeformNet’ like model and a 3D UNet. Compared to the 3D UNet, Image2Flow produces smooth, anatomically correct meshes with well-defined flat inlets and outlets and without misclassified islands.

This is also corroborated quantitatively (Table 1), with Image2Flow segmentations having significantly better HD scores than the 3D UNet (6.69 [5.95–7.30] vs. 9.87 [7.60–15.70], p = 0.003). It should be noted that ’MeshDeformNet’ also produced smooth meshes that outperformed the 3D UNet in terms of HD score (p = 0.0002). However, Image2Flow had a significantly higher Dice score compared to the ’MeshDeformNet’ (0.91 [0.86–0.92] vs. 0.88 [0.84–0.90], p = 0.004).

**Table 1:** Segmentation metrics evaluating Image2Flow, ‘MeshDeformNet’ and the 3D UNet compared to the ground truth.

Metric	Image2Flow	’MeshDeformNet’	3D UNet
**Dice (↑)**	0.91 (0.86–0.92)**	0.88 (0.84–0.90)	0.89 (0.84–0.90)
**ASSD (mm) (↓)**	1.53 (1.23–1.78)	1.38 (1.12–1.73)	1.47 (1.34–1.52)
**HD (mm) (↓)**	6.69 (5.96–7.30)*	6.99 (5.35–7.79)***	9.87 (7.60–15.70)

* Indicates statistical significance comparing Image2Flow to UNet.

** indicates statistical significance comparing Image2Flow to ‘MeshDeformNet’.

*** indicates statistical significance comparing ‘MeshDeformNet’ to Unet.

↑ denotes higher values are superior, and ↓ denotes lower values are superior.

### CFD accuracy

#### Pressure and velocity error

**Fig 4:**
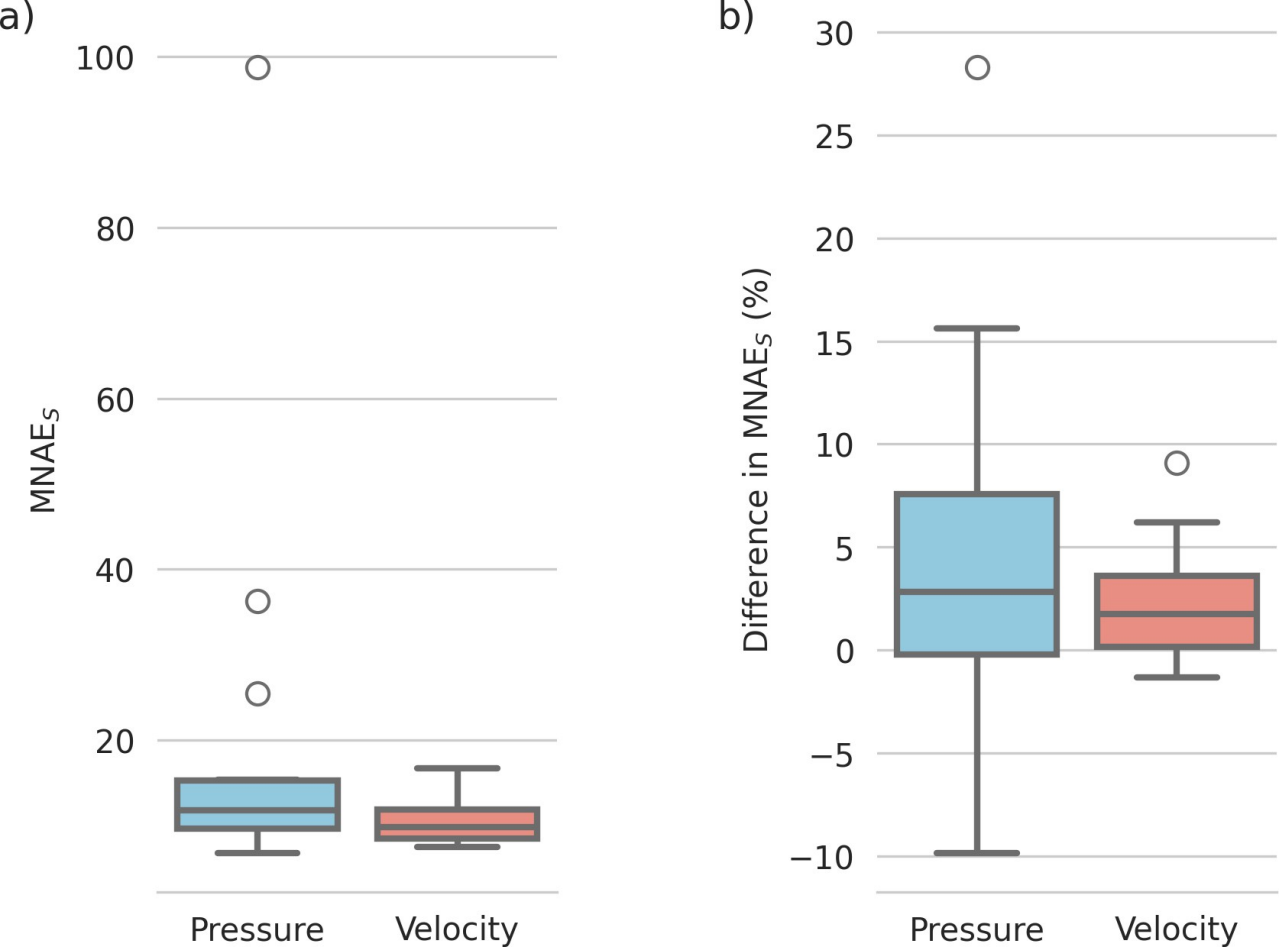
(A) MNAE_S_ values of the Image2Flow predictions compared to the ground truth on the test set (n = 15) for pressure and velocity. (B) Difference in MNAE_S_ values between CFD_I2F_ and CFD_DL-seg_.

Subject-level MNAE_s_ (CFD_I2F_ vs. CFD_GT_) for pressure and velocity magnitude are shown in Fig 4A, with median errors of 11.75% for pressure and 9.90% for velocity magnitude (see S2 Fig for separate x-y-z velocity errors).

**Fig 5:**
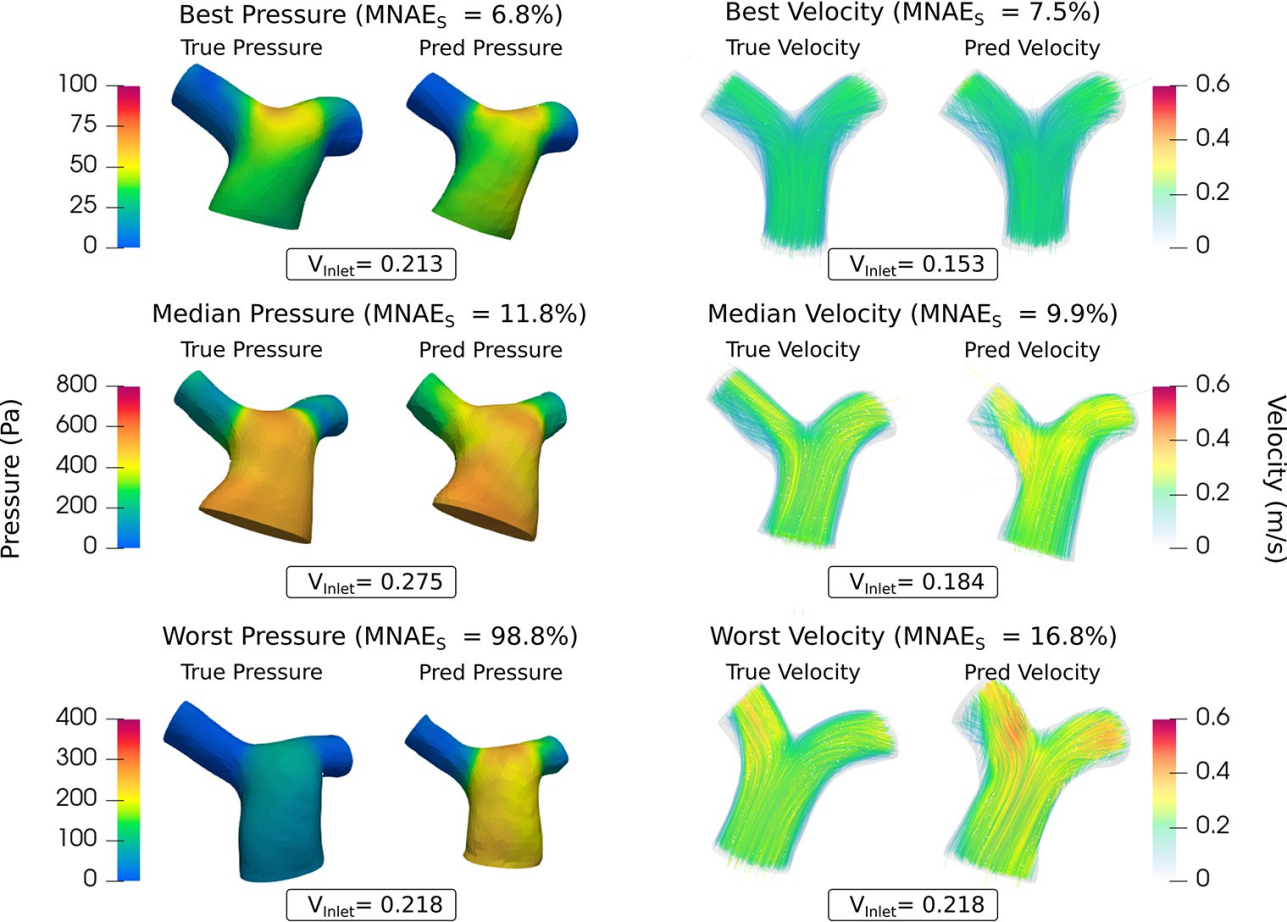
The best, median and worst blood pressure, and velocity predictions of Image2Flow by MNAE_s_. The size and positioning between the true and predicted meshes are to scale.

Fig 5 shows the best, median, and worst pressure, and velocity magnitude predictions. It should be noted that the worst pressure case was a significant outlier (as shown in Fig 4A) with most subjects having errors of <20%, with conserved pressure distributions and velocity streamline patterns.

**Fig 6:**
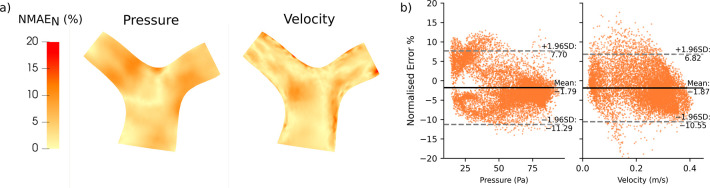
The distribution of node-wise error (MNAE_N_) of the test set (n = 15) projected onto the template pulmonary artery volume-mesh. (A) Distribution of error across the cross-section of the pulmonary artery, (B) Bland-Altman analysis of the pressure and velocity magnitude errors.

Fig 6A visualizes the distribution of node errors across all test subjects for pressure and velocity. Errors were not higher in any specific location (e.g. the bifurcation), demonstrating that Image2Flow CFD predictions have no spatially localized biases. Across the whole pulmonary artery, Bland-Altman plots (Fig 6B) do show a small but significant bias (p < .0001) in pressure (-1.79%) and velocity predictions (-1.87%). Similarly, there are minimal but significant (p < .0001) biases in the x-y-z velocity components of 2.14%, -2.56% and -0.38%, respectively (see S3 Fig).

#### Effect of varying inlet velocity

**Fig 7:**
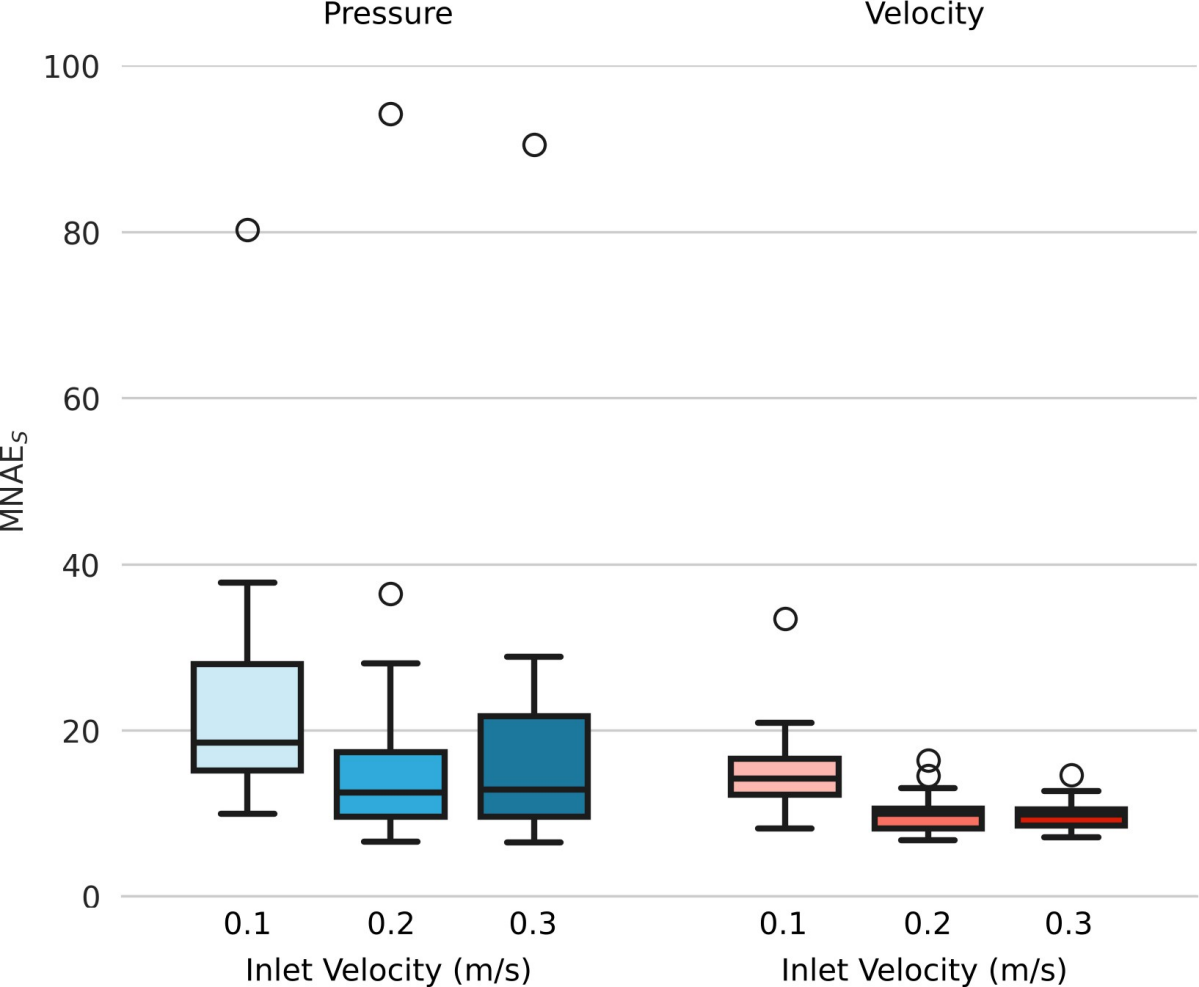
MNAE_S_ values of the Image2Flow pressure and velocity magnitude predictions for different inlet velocities (0.1, 0.2, and 0.3m/s).

Fig 7 shows pressure and velocity magnitude MNAE_s_ for inlet velocities of 0.1, 0.2, and 0.3m/s. For both pressure and velocity, MNAE_s_ values were significantly higher when V_inlet_ = 0.1m/s, compared to V_inlet_ = 0.3m/s (p<0.01). Comparing V_inlet_ = 0.1m/s with 0.2m/s, there was a trend towards a significant difference for pressure MNAE_s_ (p = 0.07), and a significant difference in velocity (p = 0.029). However, there were no significant differences in pressure or velocity MNAE_s_ between V_inlet_ = 0.2m/s and V_inlet_ = 0.3m/s (p > 0.52).

**Fig 8:**
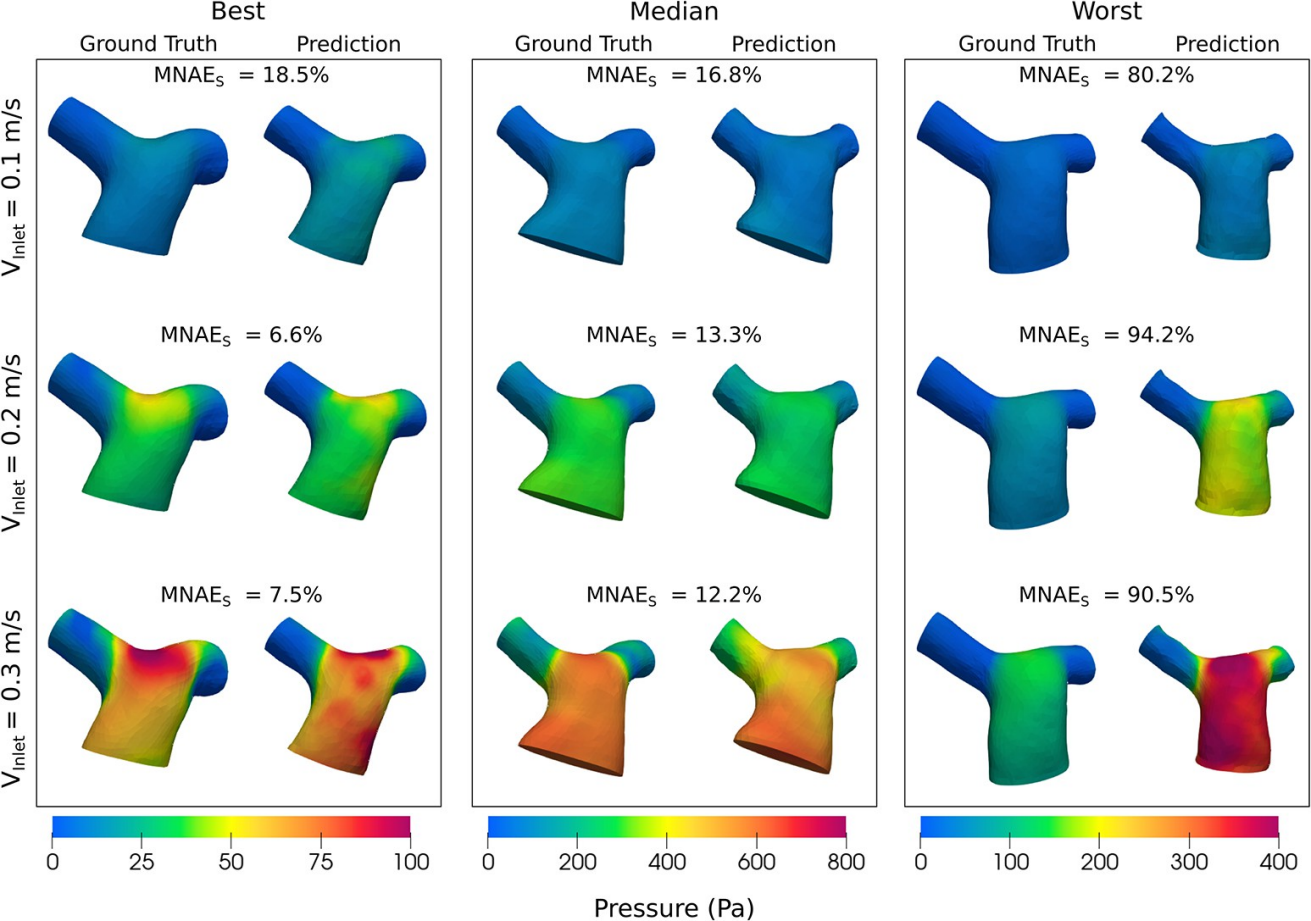
The best, median and worst blood pressure predictions of Image2Flow with varying input inlet velocity (0.1–0.3m/s). The size and positioning between the true and predicted meshes are to scale.

**Fig 9:**
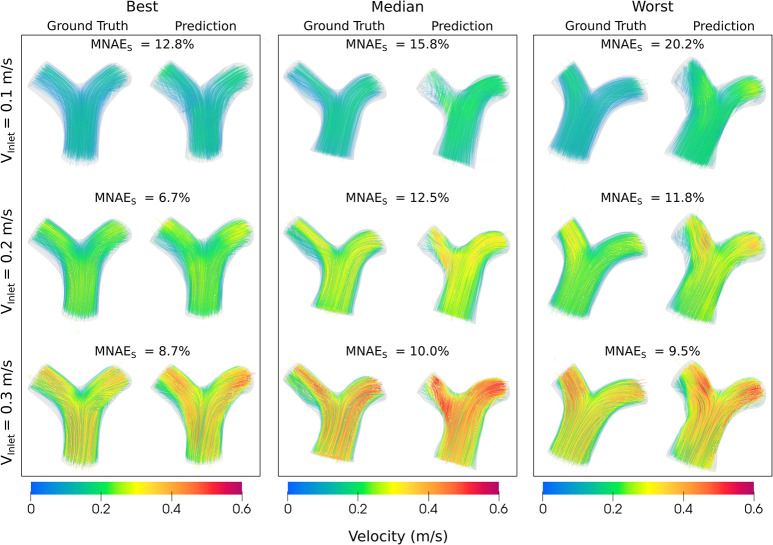
The best, median and worst blood velocity predictions of Image2Flow with varying input inlet velocity (0.1–0.3m/s). The size and positioning between the true and predicted meshes are to scale.

Figs 8 and 9 show the pressure and velocity predictions in the best, median and worst cases shown in Fig 5, for the three fixed inlet velocities 0.1, 0.2 and 0.3m/s. Both Figs 8 and 9 show that Image2Flow can generalize to different inlet velocities for the same geometries, with the pressure and velocity scaling with increased input inlet velocity.

#### Comparison with conventional CFD

**Fig 10:**
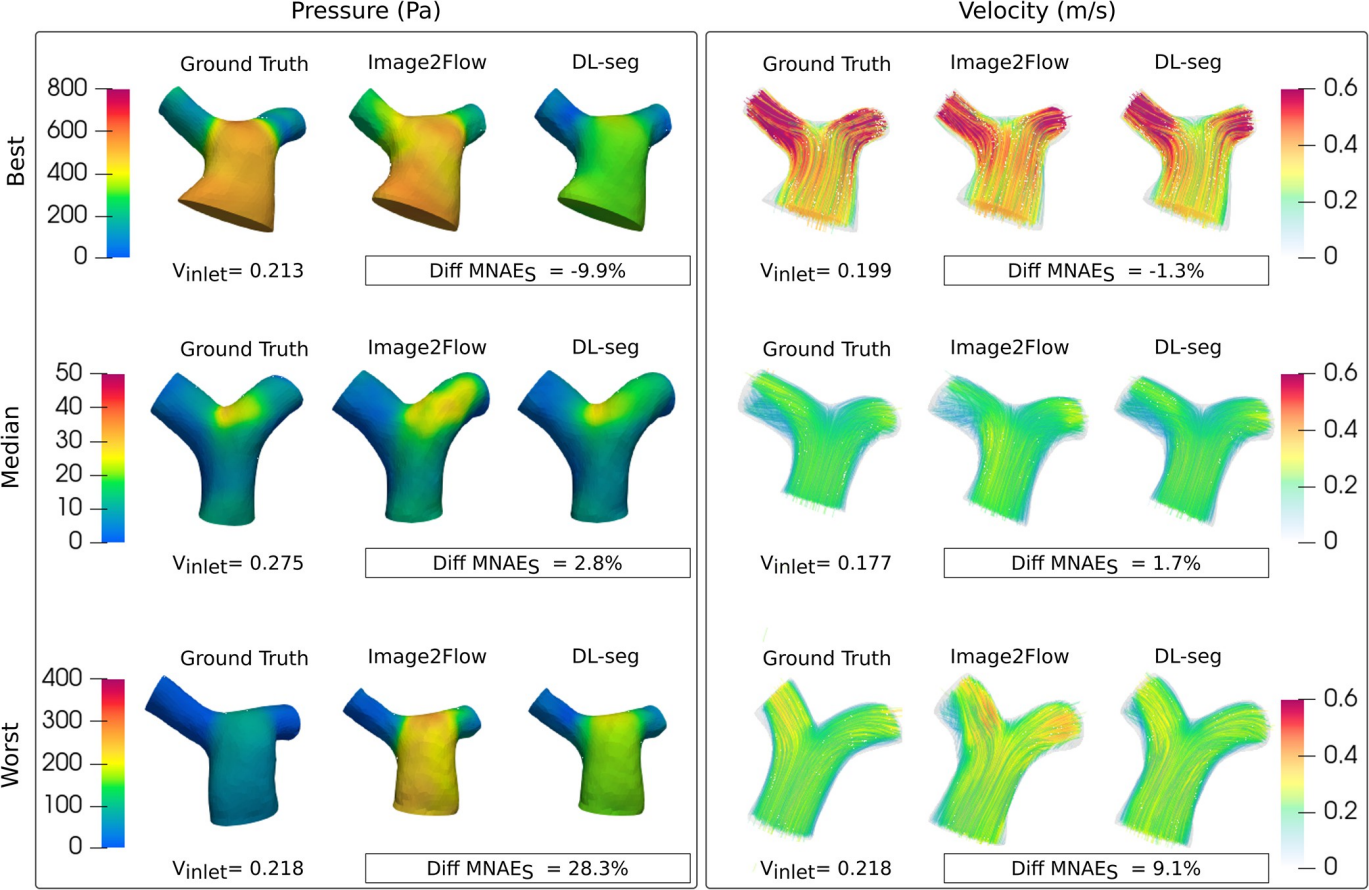
Comparison between Image2Flow CFD prediction and conventional CFD on the same geometry.

Fig 4B shows the subject level differences in MNAE_S_ between CFD_I2F_ and CFD_DL-seg_ pressure and velocity estimates. For pressure, it can be seen there is a large range of difference values (negative: CFD_I2F_ outperforms CFD_DL-seg_, positive: CFD_DL-seg_ outperforms CFD_I2F_) with a median value of 2.8%, which trended towards significance (p = 0.0535). For velocity magnitude, the range of difference values was narrower, with only a few cases where CFD_I2F_ outperformed CFD_DL-seg_ (negative values)–see Supporting Information S1 Table for separate velocity component results. Thus, the median difference in velocity MNAE_S_ of 1.7%, was statistically significant (p = 0.0051) and showed that CFD_DL-seg_ outperformed CFD_I2F_. These results can be appreciated in Fig 10, which shows the best (CFD_I2F_ outperforms CFD_DL-seg_), median, and worst (CFD_DL-seg_ outperforms CFD_I2F_) cases.

## Discussion

To our knowledge, this is the first study that uses a graph-based DL model to efficiently segment and estimate flow fields in the pulmonary arteries directly from 3D whole heart data. The main findings of this study were: i) Image2Flow can generate highly accurate pulmonary artery volume-meshes directly from cardiac MRI data, and ii) Image2Flow can also robustly estimate pressure and velocity at mesh vertices with patient-specific inlet velocities. The main benefit of Image2Flow is rapid inference (~330ms), which is approximately 5,000x faster than manual processing and conventional CFD simulation. We believe the ability to perform rapid CFD evaluation could aid with clinical translation of hemodynamic simulation in structural and congenital heart disease.

### Segmentation accuracy

Image2Flow builds on previous work that has shown that graph convolutional neural networks (e.g. MeshDeformNet) can generate surface-meshes with excellent segmentation accuracy. However, unlike previous surface-mesh based approaches, Image2Flow also estimates pressure and flow, and this requires the generation of a volume-mesh. To achieve this, we employed a modified network architecture and a volumetric template. We also utilized several novel losses that ensured the final volume-mesh had regularly shaped and sized cells (edge length deviation loss and aspect ratio loss), flat inlet and outlets (cap co-planar loss), and a smooth surface (surface edge length deviation loss). This resulted in a mesh that required no further processing (e.g. clipping). As with MeshDeformNet, Image2Flow performed better than a traditional 3D UNet, producing more anatomically consistent segmentations with flat inlet and outlets. This was possibly because mesh-based methods include an anatomical prior in the form of the template mesh. Furthermore, Image2Flow slightly outperformed our ’MeshDeformNet’ implementation in terms of Dice score, with no statistically significant differences in HD or ASSD. This demonstrates that generating volume meshes (rather than surface-meshes) does not have a deleterious impact on segmentation accuracy and may even improve robustness.

### CFD comparison

Image2Flow was able to robustly estimate pressure and flow at each mesh vertex, with no spatially localized systematic biases in pressure or flow. Importantly, Image2Flow was able to take in a specific inlet velocity and thus, produce a more patient-specific simulation. However, compared to previous DL-CFD methods, Image2Flow did have higher node-wise errors. Pajaziti et al. presented a DL-CFD model for the aorta that achieved MNAEs of ~6% for pressure and ~4% for velocity, while Li et al. achieved MNAEs of ~2–3% for both pressure and velocity in the coronary arteries [19,23]. Factors contributing to lower errors in previous models were i) they estimate pressure and velocity from pre-derived anatomy (shape descriptors or point clouds) and ii) they use the same the inlet velocities for all anatomies. On the other hand, Image2Flow attempts simultaneous segmentation and flow field estimation with variable velocity inlet conditions, which is a much more complex task. Another potential factor is the smaller amount of training data used in our study compared to previous studies. This was primarily because we were unable to easily produce synthetic data for training. Pajaziti et al. used 3000 synthetic datasets generated from a shape model in their study, which is 20 times more data than used in this paper [19]. Unfortunately, generating such datasets is challenging in our approach because synthetic images, rather than meshes, must be produced. Recently, generative adversarial models have been shown to effectively produce paired synthetic image and mesh-segmentation data [30]. We believe that such an approach could be used to generate larger training datasets and potentially improve the accuracy of Image2Flow.

One important aspect of Image2Flow is the ability to take an individualized inlet velocity as an input. To achieve this, we trained Image2Flow with multiple random velocities per anatomy, allowing more generalization to a range of inlet flow conditions. The ability to specify inlet velocities is vital in making these models truly patient specific and we have shown that Image2Flow has the potential to estimate personalized flow fields. In addition, we were also able to show that increasing the inputted inlet velocity resulted in robust predictions of increased pressure and flow. However, percentage error rates for both pressure and velocity were higher for the 0.1m/s simulations compared to the higher inlet velocity simulations, with two possible explanations. Firstly, Image2Flow was trained with inlet velocities sampled from a normal distribution of 0.2±0.05m/s, and therefore, only 2.5% of velocities used for training were ≤0.1m/s. This lack of training examples could explain poor generalization at lower inlet velocities, but this should also result in worse errors at 0.3m/s, which was not seen. Thus, poor generalization to slight out-of-distribution data cannot explain the greater errors at 0.1m/s. The second potential cause of increased errors is the normalization used to calculate MNAE_s_, which amplifies errors when the mesh pressure and velocities are low (as one would expect with lower inlet velocities). Nevertheless, the errors are still small and demonstrate that Image2Flow can be used for a wide range of velocities.

A potentially significant issue with Image2Flow is the entanglement of segmentation and flow field estimation. To investigate this issue, we also performed conventional CFD on the segmentations produced by Image2Flow. The subject-level difference in the error provided insight into whether errors were derived from inaccurate segmentation or CFD prediction. For velocity, we found errors were slightly (but statistically) lower for CFD performed on Image2Flow segmentations compared to the direct Image2Flow estimations. This suggests there was an additional error due to the CFD prediction performed by Image2Flow. However, this additional error represented a relatively small component of the total Image2Flow velocity error, which implies that geometric errors are more important. For pressure, the picture was more complicated. In some cases, Image2Flow outperformed CFD performed on Image2Flow segmentations, as shown by a negative difference MNAE_s_. This suggests that in some cases the model may learn to correct for geometric errors in anatomy, when estimating the final pressure. On the other hand, the opposite was true for other cases with Image2Flow performing worse suggesting a failure in the pressure estimation. Thus, for pressure, there is a complex entanglement of segmentation and pressure errors, and the origin of these errors requires further study if this proof-of-concept approach is to evolve into a clinical tool. In addition, expanding the training dataset and incorporating a broader range of anatomical variations could potentially improve the model’s capacity to learn more complex relationships between geometry and hemodynamics. Nevertheless, Image2Flow does provide relatively accurate measures of hemodynamics in a fraction of the time of conventional means.

### Potential clinical applications

Image2Flow provides rapid segmentation and efficient hemodynamic computation, and we believe that this has significant potential in the clinical environment. We envisage a ’one-button CFD’ approach performed by clinicians with no computational or engineering background as part of regular reporting of cardiovascular MRI studies. Routine, non-invasive estimation of hemodynamics should result in better identification of patients requiring intervention and, thus, potentially improved outcomes. Interestingly, we demonstrated that the user can triple the inlet velocity with robust estimation of pressure and velocity, potentially enabling prediction of exercise hemodynamics. As many thresholds for structural interventions are based on pressure or velocity measured during exercise, our method has great potential in the clinical environment. However, this is a proof-of-concept study, and further improvements are required prior to any clinical validation (particularly the inclusion of time-varying flow fields—see limitations).

### Limitations

The main limitation of this study is that our model is trained only to predict simplistic CFD with identical outlet boundary conditions and steady-state conditions for all patients. Morbiducci et al. [31] demonstrated that idealized boundary conditions can yield misleading representations of hemodynamics, emphasizing the necessity for patient-specific parameters to simulate realistic blood flow patterns [32]. In our study, we did use individualized inlet velocities, and these could easily be acquired from patient-specific flow profiles derived from phase-contrast MRI data. However, we still used 0Pa outlet conditions for both pulmonary arteries and in all patients. This is non-physiological, as pulmonary vascular resistance differs between subjects and between lungs, but was done to reduce the complexity of the problem. Nonetheless, we have shown that individualized inlet velocities can be incorporated into our model and outlet lumped parameters could be included in a similar way. Thus, we believe that Image2Flow represents a framework that could be easily modified to incorporate comprehensive patient-specific boundary conditions. Another important limitation of our current Image2Flow framework was the use of steady-state flows rather than more realistic dynamic flows. Once again, this was done to simplify the problem in this proof-of-concept study. Nevertheless, this should be included in future modifications of Image2Flow and will require the development of novel time-varying graph architectures.

A further limitation of our approach is that image acquisition was similar in all cases. This means that we were unable to investigate the effect of spatial resolution on segmentation accuracy or pressure and velocity estimation. In addition, we did not include infants with high heart rates and our method may not work as well in these patients. In addition, we have not demonstrated that our method works with recent whole-heart approaches that utilize blood pool contrast agents such as Ferumoxytol. Thus, it is vital that future studies demonstrate that our approach is generalizable to different resolutions, patient sizes, and contrast.

Image2Flow also faces constraints on the number of nodes it can predict within the volume-meshes, which is currently limited to around 11,000 nodes due to GPU memory constraints (as also highlighted by Kong et al. [16]). Given the high-resolution volume meshes necessary for accurate blood flow calculations in CFD simulations, our node-wise evaluation is restricted to comparing low-resolution CFD predictions with ground truth results interpolated onto low-resolution volume meshes. Thus, in the future, some effort will be required to find more memory-efficient methods of performing these graph-based computations.

## Conclusion

Our proof-of-concept study introduces Image2Flow, a hybrid image and graph deep learning model for efficient pulmonary artery segmentation and flow field estimation from 3D whole heart data. Evaluated on 15 test cases, Image2Flow demonstrated superior segmentation accuracy compared to 3D UNet and MeshDeformNet, directly reconstructing anatomically correct volume meshes. The model exhibited robust pressure and velocity estimation shown through node-wise analysis and comparison with conventional CFD.

Image2Flow excels in efficiency, producing flow fields over 5000x faster than conventional methods in a single pass, without requiring clinical and engineering expertise. This speed makes it promising for integration into a clinical setting, allowing swift patient evaluation for intervention and improved treatment outcome predictions. However, limitations and challenges remain, including the accuracy of point-point correspondence between nodes and the need for patient-specific parameters for more realistic CFD simulations.

## Supporting information

S1 Fig Sensitivity analysis. Mesh sensitivity study conducted using two random pulmonary artery shapes sourced from private and public datasets. Volume meshes of approximately 200,000, 400,000, 600,000, and 800,000 cells were compared for each shape under identical CFD simulation boundary conditions, focusing on centerline pressure and velocity values. The analysis concluded with a decision to use 500,000 cells in the mesh to maintain a balance between accuracy, computation time, and memory usage.
(TIF)

S2 Fig (A) MNAE_S_ values of the Image2Flow predictions compared to the ground truth on the test set (n = 15) for pressure and velocity magnitude and x-y-z components. (B) Difference in MNAE_S_ values between CFD_I2F_ and CFD_DL-seg_.
(TIF)

S3 Fig CFD prediction error distribution across the pulmonary artery. The distribution of node-wise error (MNAE_N_) of the test set (n = 15) projected onto the template pulmonary artery volume-mesh. (A) Distribution of error across the cross-section of the pulmonary artery, (B) Bland-Altman analysis of the pressure and velocity magnitude errors, (C) Bland-Altman analysis of the errors of each of the x-y-z components of velocity.
(TIF)

S1 Table Subject-level CFD prediction error for Image2Flow compared to the ground truth CFD interpolated onto point-point correspondent meshes. (XLSX)
