# Supplementary figures and images for: Image2Flow: A proof-of-concept hybrid image and graph convolutional neural network for rapid patient-specific pulmonary artery segmentation and CFD flow field calculation from 3D cardiac MRI data
Source: PLoS Comput Biol. 2024 Jun 20;20(6):e1012231. doi: 10.1371/journal.pcbi.1012231 (PMC11218942; doi:10.1371/journal.pcbi.1012231)

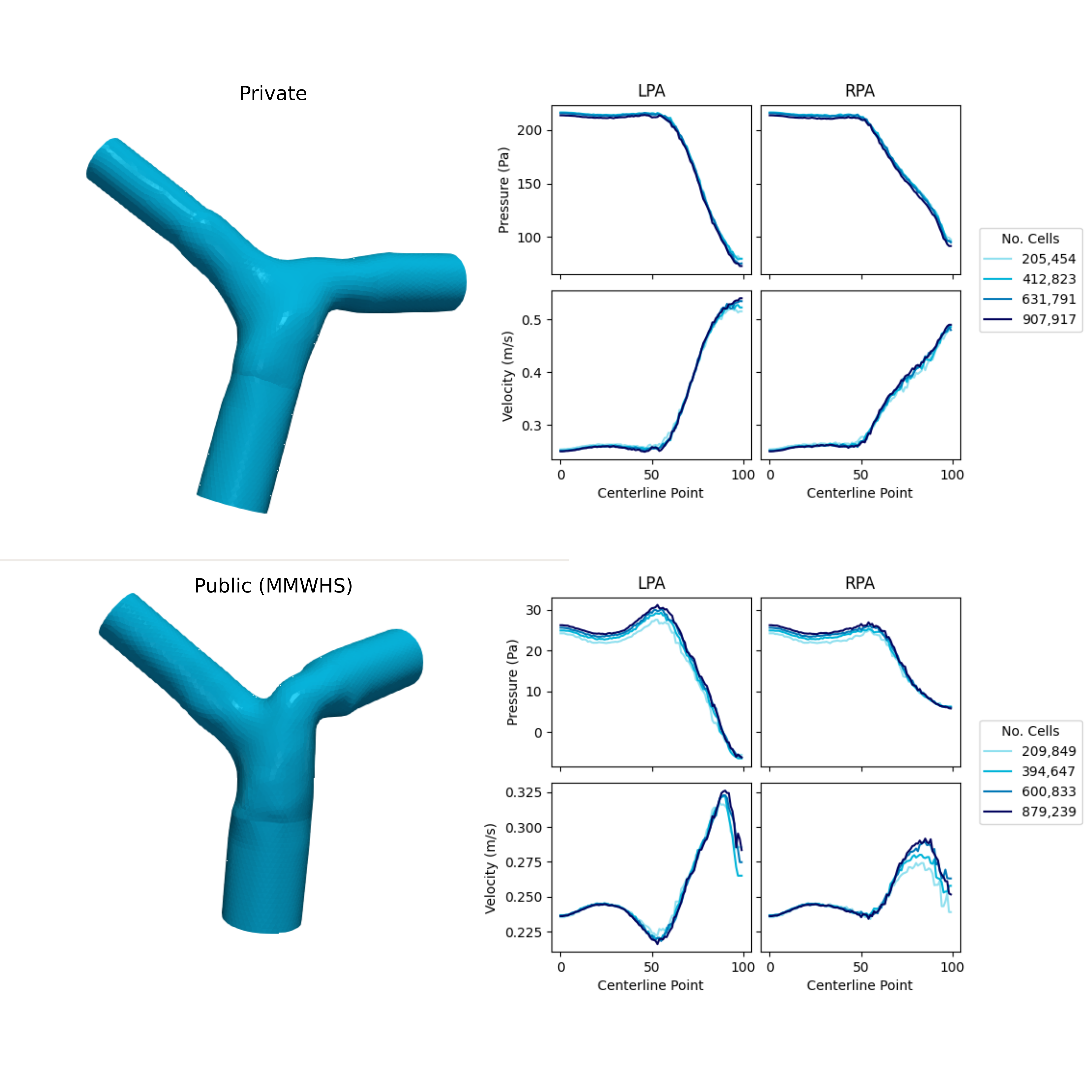

Supplement: S1 Fig — Mesh sensitivity study conducted using two random pulmonary artery shapes sourced from private and public datasets. Volume meshes of approximately 200,000, 400,000, 600,000, and 800,000 cells were compared for each shape under identical CFD simulation boundary conditions, focusing on centerline pressure and velocity values. The analysis concluded with a decision to use 500,000 cells in the mesh to maintain a balance between accuracy, computation time, and memory usage. (TIF) [file pcbi.1012231.s001.tif]

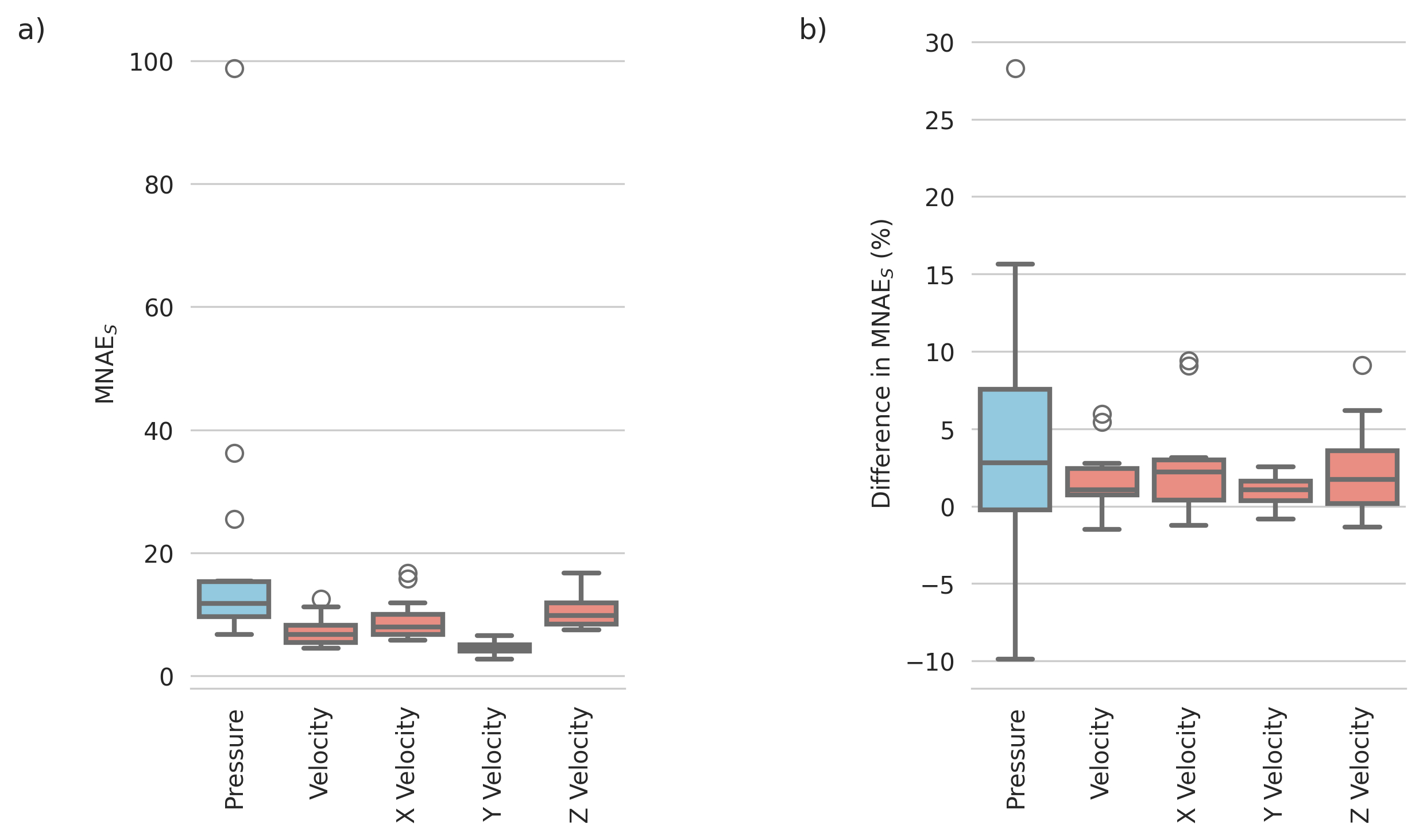

Supplement: S2 Fig — (A) MNAES values of the Image2Flow predictions compared to the ground truth on the test set (n = 15) for pressure and velocity magnitude and x-y-z components. (B) Difference in MNAES values between CFDI2F and CFDDL-seg. (TIF) [file pcbi.1012231.s002.tif]

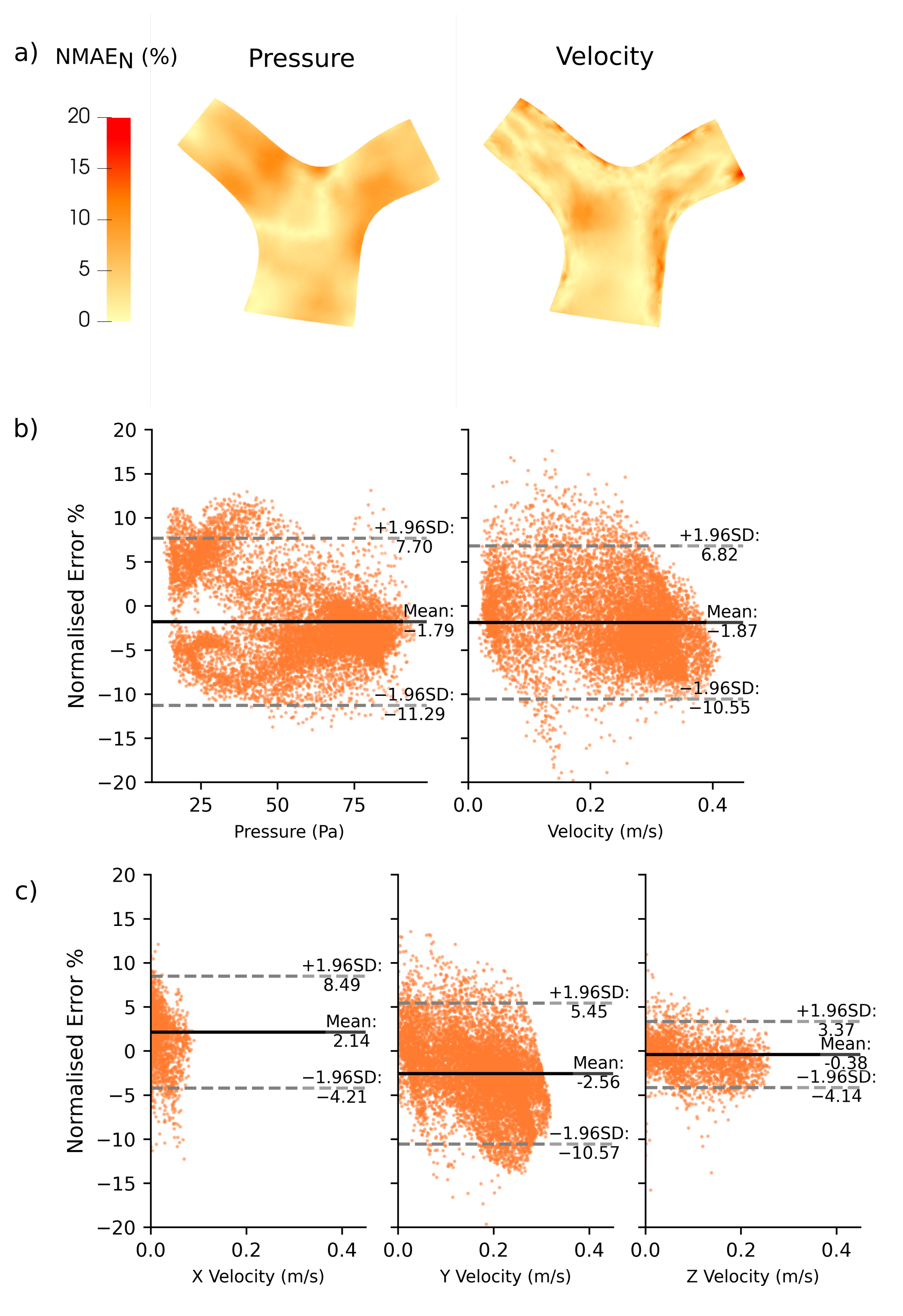

Supplement: S3 Fig — The distribution of node-wise error (MNAEN) of the test set (n = 15) projected onto the template pulmonary artery volume-mesh. (A) Distribution of error across the cross-section of the pulmonary artery, (B) Bland-Altman analysis of the pressure and velocity magnitude errors, (C) Bland-Altman analysis of the errors of each of the x-y-z components of velocity. (TIF) [file pcbi.1012231.s003.tif]
